# Honors to Medical Men

**Published:** 1860-11

**Authors:** 


HONORS TO MEDICAL MEN.—Drs. Ricord and Jules Cloquet have recently been promoted to the rank of Commanders of the Legion of Honor, a body which, in the time of the first Napoleon, numbered but a few hundred. At the present day the members of the Legion amount to many thousands.

